# Simulation-Based X-Ray Spectrum Optimization for Dose Enhancement in X-Ray-Induced Photodynamic Therapy with NaLuF_4_:20% Gd, 15% Tb^3+^ Nanocrystals

**DOI:** 10.3390/bioengineering13030316

**Published:** 2026-03-10

**Authors:** Wangyang Li, Peng Gao, Ruijing Li, Tianshuai Liu, Zhongfei Wang, Wenli Zhang, Hongbing Lu, Yang Liu, Junyan Rong

**Affiliations:** 1 School of Biomedical Engineering, Fourth Military Medical University, No. 169 Changle West Road, Xi’an 710032, China; liwangyang1995@163.com (W.L.); xiangzhiwanli@163.com (P.G.); liruijing1025@163.com (R.L.); liutianshuai91@126.com (T.L.); wenlizhang1121@163.com (W.Z.); luhb@fmmu.edu.cn (H.L.); 2 Shaanxi Provincial Key Laboratory of Bioelectromagnetic Detection and Intelligent Perception, No. 169 Changle West Road, Xi’an 710032, China; 3 Department of Radiation Oncology, Xijing Hospital, Fourth Military Medical University, No. 127 Changle West Road, Xi’an 710032, China; sean_wong@163.com

**Keywords:** dose enhancement ratio, Monte Carlo, tumor growth inhibition, X-ray-induced photodynamic therapy

## Abstract

**Context:** X-ray-induced photodynamic therapy (X-PDT) is an emerging modality for deep-tumor treatment, leveraging radiosensitizing nanoprobes to enhance therapeutic effects. **Problem:** However, the efficacy of X-PDT is critically dependent on the incident X-ray energy, and the selection of an optimal spectrum remains a significant clinical challenge. **Motivation:** A systematic approach to optimizing X-ray energy is crucial for maximizing dose enhancement and translating X-PDT’s potential into effective clinical outcomes. **Aim:** This study therefore aims to identify the optimal X-ray energy for X-PDT by systematically evaluating the dose enhancement provided by nanoprobes across a range of clinically relevant energies. **Proposed Methodology:** We developed a Geant4-based Monte Carlo model to simulate the absorbed dose in a tumor phantom, both with and without X-PDT nanoprobes, under various X-ray energies (20–160 kVp). The dose enhancement ratio (DER) was calculated to quantify the sensitization effect. To validate our model, the simulated dose data were directly correlated with in *vivo* experimental tumor growth inhibition (TGI) rates. **Main Results:** The absorbed dose peaks at shallow depths (<1.0 mm), and the 60 kVp X-ray beam yielded the highest peak dose and the maximum DER. Furthermore, the absorbed dose calculated in the model incorporating nanoprobes showed a stronger correlation with experimental TGI than the model without them, confirming the simulation’s predictive value. **Conclusions:** There is an optimized X-ray excitation energy value for maximizing the therapeutic effect for X-PDT. The simulation method provides a potential way to optimize the treatment protocols for X-PDT. But accurate simulation needs to be carried out in combination with more biological modeling.

## 1. Introduction

X-ray-induced photodynamic therapy (X-PDT) is a combination of photodynamic therapy (PDT) and radiation therapy (RT) [1,2,3] and has shown great potential because of its unique mechanism [4,5,6,7]. X-PDT uses X-rays as an excitation source for nanoparticles (NPs) with complex physical and chemical interactions. The nanoscale scintillator absorbs high-energy X-rays and emits lower-energy ultraviolet and visible photons. Subsequently, it activates the nearby photosensitive molecules, initiating photodynamic therapy [8,9]. The method not only overcomes the limitations of conventional PDT in terms of depth of penetration but also retains the selective killing effect of RT on tumor tissues [10,11]. Owing to its distinctive advantages and tumor inhibition performance, X-PDT has received extensive attention and has been developed as a promising antitumor treatment [12,13]. This study will focus on the initial physical stage of radiation sensitization effect. We explore the optimal irradiation conditions by simulating the local deposition of physical dose, and in this model, the absorbed dose is used as a direct characterization of the initial reactive oxygen species (ROS) production. This method is based on the core physical-chemical assumption that the initial production of ROS is directly related to the local absorbed dose, enabling us to optimize the irradiation parameters from a fundamental physics perspective.

In practice, the precise control and optimization of X-ray energy and dose are crucial for maximizing tumor destruction while minimizing damage to surrounding normal tissues during radiotherapy or X-PDT [9,14,15,16,17]. Notably, X-ray energy has a potentially important impact on the therapeutic outcomes and may involve underexplored mechanisms of action [18,19]. However, the selection of irradiation conditions, including X-ray energy for X-PDT, faces challenges. The existing X-ray excitation energy range is often broad, and systematic comparative studies are scarce [15,20]; this results in a lack of clear guidance for selecting a suitable energy range. In addition, problems, such as long experimental periods, high resource consumption and difficulty in fully covering experimental conditions, increase the difficulty of comparative research. Moreover, due to various complex influencing factors in the experiments, such as the state of animal models and X-ray stability, the contribution of each factor is difficult to accurately separate and quantify; this causes slow progress in the study of synergistic mechanisms [18,21]. Therefore, the development of an efficient and accurate optimization method to determine the irradiation conditions in X-PDT is highly important for promoting the clinical application of this therapy.

The introduction of nanoparticle systems with high-Z elements and photosensitizers adds a new dimension to this process. Nanosystems sensitive to X-rays can efficiently target the tumor site and strengthen the synergistic effect of RT and PDT while increasing the X-ray absorbed dose. Among them, the use of X-ray energy plays a crucial role as follows [15]. The use of X-rays can affect the photoelectric effect probability, X-ray penetration depth, dose distribution, luminescent activation efficiency of the photosensitizer [22,23,24,25] and the therapeutic effect of X-PDT [21]. Thus, the precise selection of the energy range and in-depth investigation of the enhancement mechanisms are important for revealing the X-PDT effect.

Given the intricate influence of X-ray energy levels and the complexities inherent in experimental research, Monte Carlo (MC) simulation is used as a potent tool for studying the dose-sensitizing effect of kV-level X-ray energy on X-PDT to optimize the excitation X-ray energies. Most current research on modeling and simulation within the field of X-PDT has focused predominantly on the interaction between the ionizing radiation and the light generated by nanoscintillators and photosensitizers; however, these simulations have been conducted under specific irradiation conditions with X-rays of 80 kV, 220 kV or 6 MV [26,27,28]. Our study expands the scope of energy to a broader range of kV-level X-rays to systematically investigate the energy-dependent physical dose radiosensitization effect. The primary objective of this study is to computationally isolate and quantify the physical foundation of the radiosensitization mechanism. Our core hypothesis is that the physical dose enhancement acts as a robust proxy for the initial yield of ROS. Therefore, we posit that the calculated Dose Enhancement Ratio (DER) can be interpreted as an ROS Production Enhancement Factor, making it a strong predictor for the efficacy of the radiolysis-driven therapeutic effect. By doing this, we aim to provide deeper insights into the depth effect of energy and the dose radiosensitization effect across different energy levels. Furthermore, we integrate our simulation findings with experimental data and provide a comprehensive validation of our simulation models.

In this work, the dose-sensitizing effect of kV-level X-ray energy on X-PDT is thoroughly examined using MC simulation. The simulation model and simulation setting are initially created. The results from the dose distribution, the radiosensitization effects of X-PDT with different kVp X-rays and a correlation between the simulation dose and the tumor inhibition rate in actual experiments are provided. Finally, a discussion and conclusion are presented.

## 2. Materials and Methods

### 2.1. Simulation Procedure

**Figure 1:**
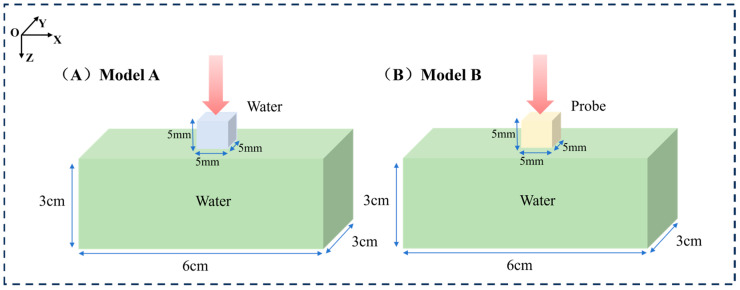
Schematic representation of the simulated models for mouse tumors. (**A**) Left: Model A consisting of a cube with a side length of 5 mm and a rectangular prism with dimensions of 6 cm, 3 cm, and 3 cm filled with water. (**B**) Right: Model B having the same shape and size as the first model with the material of NaLuF_4_:20% Gd, 15% Tb^3+^NPs@AEP-RB.

The GEANT4 toolkit (version 11.3) was used for all Monte Carlo simulations. To study the effects of different X-ray energies on absorbed doses in specific tumor models and surrounding tissues, two mouse models in which the tumor was loaded with and without nanoparticle probes were constructed. The irradiated geometry is shown in Figure 1. The first model (Model A) consists of a cube with a side length of 5 mm and a rectangle with dimensions of 6 cm, 3 cm, and 3 cm. Model A is composed of water, and the cube is used to simulate a tumor on a mouse. The second model (Model B) has the same shape and size as the first model, but the material of the cube is replaced with NaLuF_4_:20% Gd, 15% Tb^3+^NPs@AEP-RB, at a concentration of 10 mg/mL; here, the radiosensitization effect of nanoparticles on the tumor in X-PDT is simulated, and the optimal X-ray irradiation energy is determined. It is important to note that by defining the nanoparticle-laden volume as a homogeneous material within the Geant4 framework, the increased probability of photon absorption by the high-Z elements of the nanoparticle is inherently accounted for at a microscopic, probabilistic level. This stochastic approach correctly models the average effect of self-absorption on the photon flux throughout the target volume.

**Figure 2:**
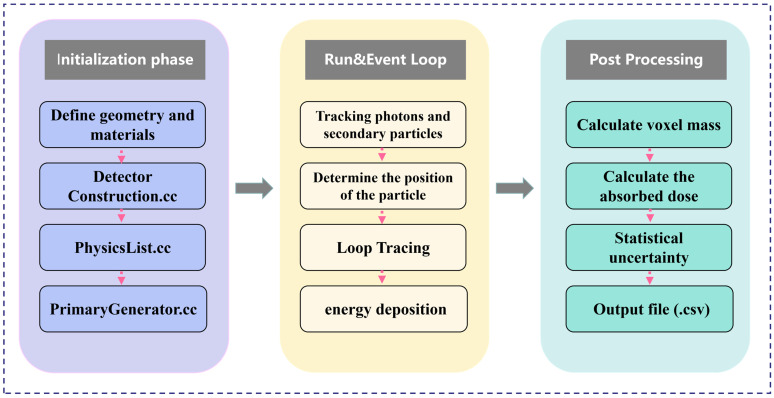
Schematic flowchart of the Geant4 simulation process.

For the two models, parallel X-ray beams with up to 10^9^ photons were cast to exactly cover the entire tumor. For a detailed analysis of the dose distribution with different X-ray energies, seven photon beams in the range of 20 kVp–160 kVp with an interval of 20 kVp were simulated; this X-ray range covers the commonly used kV X-rays. The X-ray source for this simulation was modeled based on the actual parameters of the VAREX NDI-225-22 X-ray tube (Varex Imaging, Salt Lake City, UT, USA). We simulated a multi-energy spectrum generated by a tungsten target and 0.8 mm beryllium inherent filtration, and used it as the input source for subsequent energy deposition calculations. All simulation computations were completed on a workstation equipped with an Intel(R) Xeon(R) Gold 6226R CPU (2.90 GHz, 16 cores, 32 threads). Under this configuration, the complete simulation for a single incident energy took approximately 10 h. To map the spatial distribution of energy deposition, the cubic tumor volume in all models was divided into a 3D grid of voxels, each with dimensions of 0.2 mm × 0.2 mm × 0.2 mm. This voxel size was selected as a strategic balance between achieving sufficient spatial resolution to resolve macroscopic dose gradients and maintaining a low statistical uncertainty in the scored dose for each voxel. The voxel size is large enough to capture the key dose variations, while also being able to accumulate sufficient statistical data for each voxel from 10^9^ original photons. The energy absorbed by each voxel is recorded to calculate the absorbed dose, which lays the foundation for our analysis of dose distribution characteristics and optimization of X-ray energy. To accurately simulate electromagnetic interactions, the G4EmLivermore physics list was adopted. This physics list is specifically designed for low-energy applications and can provide high-precision, data-based cross-sections for our key processes within this energy range, including photoelectric effect, Compton scattering, and Rayleigh scattering. For photons and electrons, a low secondary production cutoff value of 210 eV was set. This ensures that even very low-energy secondary particles can be accurately generated and tracked. The dose data served as a basis for analyzing the characteristics of the dose distribution at different X-ray kVps and for optimizing the X-ray energy. The entire simulation pipeline, from initial setup to final data extraction, is schematically illustrated in Figure 2. It outlines the key stages of initialization, the run and event loop, and post-processing.

**Figure 3:**
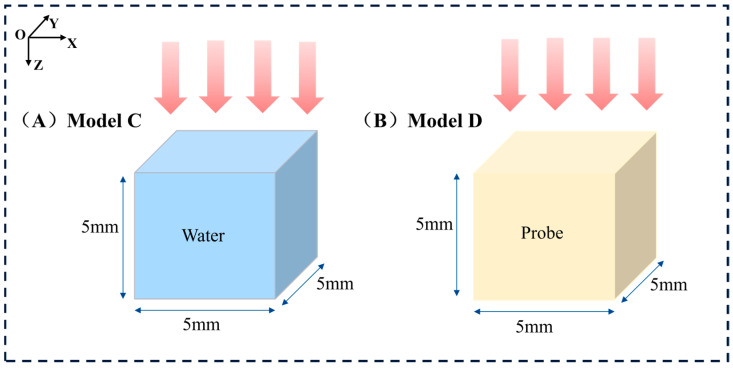
Schematic representation of the simulated tumor-only models. (**A**) Left: Model C on the left is the tumor-only model without nanoparticles. (**B**) Right: Model D is the tumor-only model with the material NaLuF_4_:20% Gd, 15% Tb^3+^NPs@AEP-RB.

Furthermore, considering that the dose distribution within the tumor region is a critical factor and that surrounding tissues may interfere with the dose distribution, we simplified the model by isolating the tumor portion for simulation to analyze the impact of the probe on the internal dose distribution within the tumor more precisely. As shown in Figure 3, the tumor-only model geometry is as follows: in Model C, a cube with a side length of 5 mm was filled with water to simulate the tumor; Model D had the same shape and size as Model C, but its material was identical to the probe material used in Model B. The simulations were performed under the same irradiation conditions as those of the previous two models. Using this simplified approach, we were able to investigate the characteristics of the internal dose distribution within the tumor more accurately. A key foundational assumption of this study is the uniform and homogeneous distribution of nanoparticles throughout the tumor volume in the relevant models (Model B and D). This is an idealization that simplifies the complex in *vivo* biodistribution, allowing for a focused analysis of the underlying radiation physics. This limitation is further discussed in Section 4.

Additionally, we analyzed the percentage depth dose (PDD) to determine the dose deposition at different depths. To evaluate the radiosensitization effect of the probe, dose enhancement ratio (DER) curves were analyzed to determine the dose enhancement achieved using nanoparticle radiosensitization and to provide deeper insights into the spatial dose characteristics and treatment efficacy. The DER [29] (Formula (1)), which is defined as the ratio of the depth dose at a point with a probe to the same point without a probe (i.e., using only water), was evaluated. Since the nanoparticles can increase the dose, the DER values were expected to increase with the addition of nanoparticles.

(1) $$\mathrm{DER}=\frac{\mathrm{Depth}\;\mathrm{dose}\;\mathrm{at}\;a\;\mathrm{point}\;\mathrm{with}\;\mathrm{addition}\;\mathrm{of}\;\mathrm{probe}}{\mathrm{Depth}\;\mathrm{dose}\;\mathrm{at}\;\mathrm{the}\;\mathrm{same}\;\mathrm{point}\;\mathrm{without}\;\mathrm{addition}\;\mathrm{of}\;\mathrm{probe}}$$

### 2.2. X-PDT in Tumor-Bearing Mice

To verify the accuracy of the simulation, the simulation results were compared with the experimental results using the tumor growth inhibition results. For the experiments, mouse mammary carcinoma 4T1 cells were obtained from the Cell Bank of the Chinese Academy of Sciences. All mice were provided by the Animal Center of the Fourth Military Medical University. All animal experiments were approved by the Animal Experiment Administration Committee of the Fourth Military Medical University and were carried out in accordance with the National Institutes of Health Guide for the Care and Use of Laboratory Animals. 4T1 tumor-bearing mice were established via the subcutaneous injection of 3 × 10^6^ 4T1 cells. When the tumor size reached 70~100 mm^3^ (near 125 mm^3^), 100 μL of 10 mg/mL NaLuF_4_:20% Gd, 15% Tb^3+^ NPs@AEP-RB solution was injected, corresponding to Model B in the simulation. After 12 h, with the exception of the control group, the tumor-bearing mice were all irradiated with a surface absorbed dose of 3 Gy and were divided into four groups, with 5 mice per group, under 40, 80, 120, and 160 kVp X-rays. During irradiation, with the exception of the tumors, the remaining mouse body was shielded by a 3 mm thick lead plate. The tumor volume was monitored after tumor formation using the commonly used Formula (2) [18]:

(2) T = 0.5 × L × W

where T is the volume of the tumor (mm^3^), and L and W are the length and width of the tumor in millimeters, respectively. The value “0.5” is derived from a large number of experimental observations and experiences and is used to correct the product of the long and short diameters to more closely approximate the actual tumor volume. The tumor growth inhibition rate (TGI) was calculated using Formula (3):

(3) $$\mathrm{TGI}\;=\;(1-\frac{V_{\mathrm{TE}}-V_{\mathrm{TS}}}{V_{\mathrm{NE}}-V_{\mathrm{NS}}})\;\times \;100\%$$

where V_TE_ and V_TS_ are the final and initial volumes of the tumor to be measured, respectively, and V_NE_ and V_NS_ are the final and initial volumes of the tumor in the control group, respectively.

## 3. Results

### 3.1. Simulated Dose Distribution with Mouse Tumors

**Figure 4:**
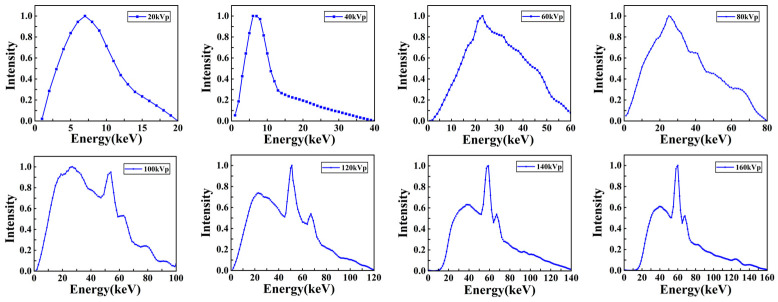
X-ray energy spectra with different kVps used in the simulation. The figures from left to right and top to bottom are as follows: 20 kVp, 40 kVp, 60 kVp, 80 kVp, 100 kVp, 120 kVp, 140 kVp, and 160 kVp.

**Figure 5:**
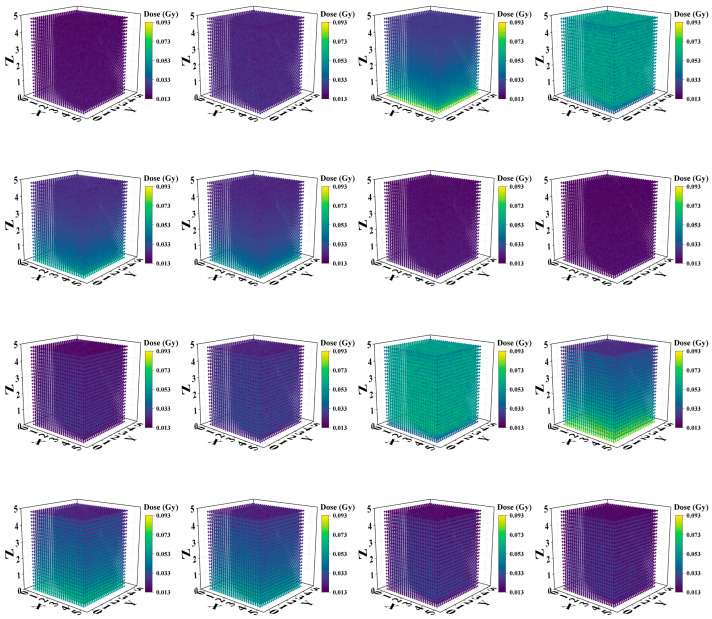
Three-dimensional (3D) dose distributions of Model A and Model B with different kVp X-rays. The first two rows are the 3D dose distributions for Model A, and the last two rows are the dose distributions for Model B. From left to right and top to bottom: 20 kVp, 40 kVp, 60 kVp, 80 kVp, 100 kVp, 120 kVp, 140 kVp, and 160 kVp.

The X-ray energy spectra of different kVp X-rays were calculated, as shown in Figure 4. These spectra were generated by accelerating and directing the incident electrons with different kV voltages on a tungsten anode; here, the tungsten target thickness was 0.2 mm, and the target angle was 20°. The filter material before the X-rays was 0.8 mm Be. The mean energies of 20–160 kVp X-rays were 8.3 keV, 11.7 keV, 29.7 keV, 33.7 keV, 40.0 keV, 44.7 keV, 57.2 keV, and 62.0 keV. The three-dimensional dose distributions of Models A and B are plotted in Figure 5.

**Figure 6:**
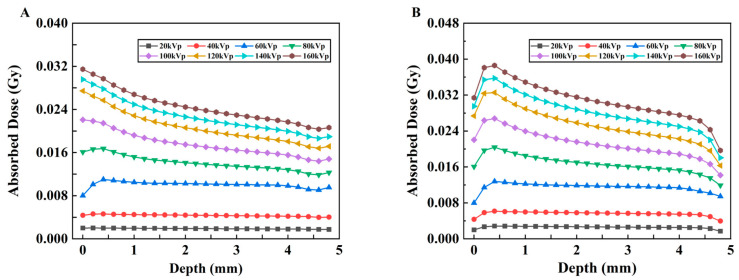
Changes in the absorbed dose vs. depth under different kVp X-rays. (**A**) Model A and (**B**) Model B.

To assess the absorbed dose as a function of depth in separate uniform media with different kVp of X-rays, the relationships between the absorbed dose and depth of X-ray in two different material models are shown in Figure 6A,B. The vertical coordinate represents the deposited absorbed dose in each voxel along the irradiation direction of the model, whereas the horizontal coordinate represents the voxel depth. The curves with low voltages smaller than 80 kVp change more smoothly than those with high voltages for both models, although these changes are not drastic. In addition, as shown in Figure 6B, with the addition of the probe, the absorbed dose at each kVp predominantly exhibited an initial increase followed by a subsequent decrease, and a minor dose buildup occurred at a depth of approximately 0.5 mm. However, this trend of change is not evident in Model A (Figure 6A), in which most values show a gradually decreasing trend with depth.

**Figure 7:**
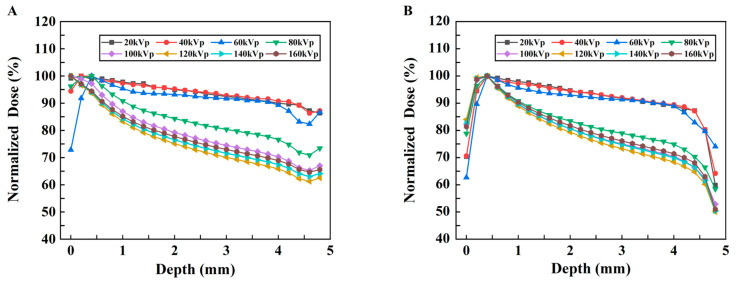
Changes in the normalized dose vs. depth under different kVp X-rays. (**A**) Model A. (**B**) Model B.

To display the dose–depth variation relationship more clearly, the PDD curves of the two models are plotted, as shown in Figure 7A,B. In Model B, with increasing depth, the PDD initially increases and then decreases. The peak value appears in the shallow layer of the model; these results indicate that its energy is gradually absorbed, resulting in a lower dose in the deeper layer.

### 3.2. Radiosensitization Effect of X-PDT

**Figure 8:**
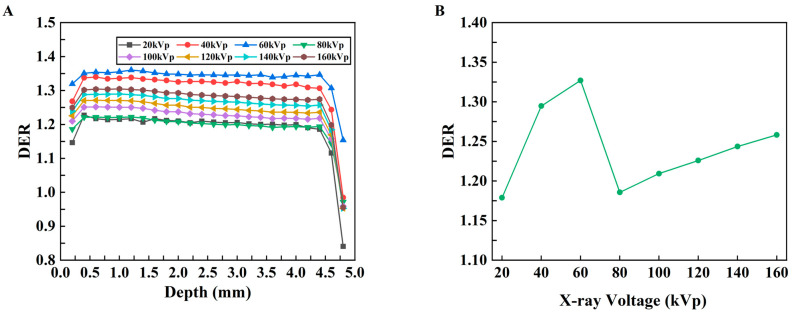
DER results for the mouse tumor model. (**A**) Changes in the DER with respect to depth under different kVp X-rays. (**B**) Changes in the DER with respect to kVp for the average dose in the whole tumor.

The relationship between DER and depth was obtained with different kVp X-rays, as shown in Figure 8A. Overall, the DER initially increases and then decreases with increasing depth for all kVp X-rays. To evaluate the influence of the probe on the mean dose enhancement in the models with different kVps, the enhancement ratio of the average dose was calculated, and the relationship between the DER of the average absorbed dose (AAD) and the kVp was obtained, as shown in Figure 8B. For every X-ray voltage, with the exception of the outermost point, the DER is greater than 1.0; these results indicate that the absorbed dose in the tumor tissue increases after the probe material is added and that the DER has the highest value at 60 kVp.

### 3.3. Tumor-Only Simulation

**Figure 9:**
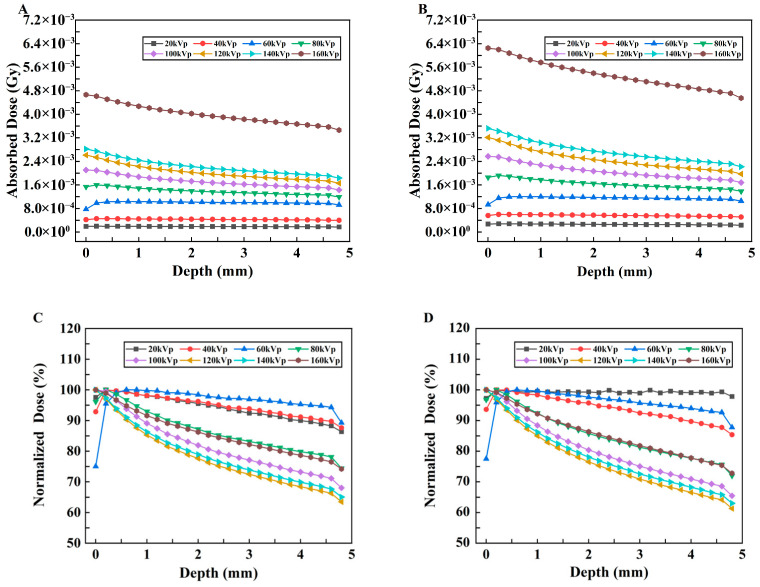
Changes in the absorbed dose with depth for Model C and Model D under different kVp X-rays and their respective PDD curves. (**A**,**B**) Changes in the absorbed dose vs. depth under different kVp X-rays for Model C and Model D, respectively. (**C**,**D**) PDD curves for Model C and Model D, respectively.

**Figure 10:**
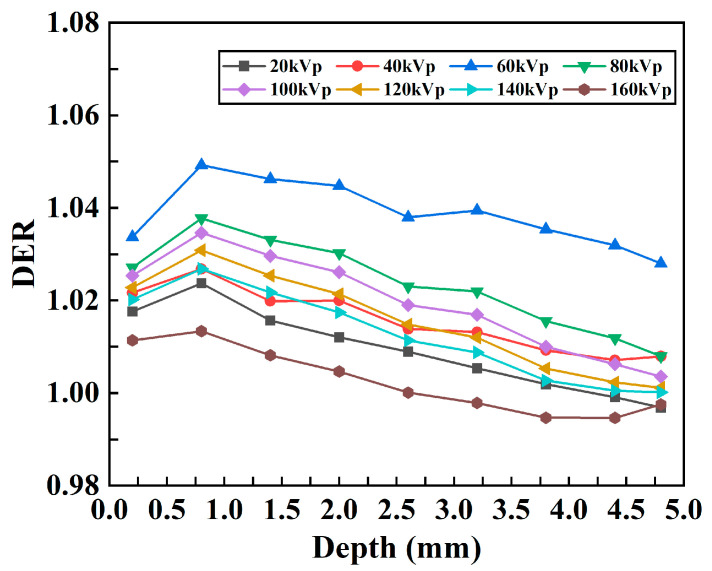
Changes in the DER with respect to depth under different kVp X-rays for the tumor-only model.

The relationships between the absorbed dose and depth of X-rays in Models C and D and the PDD curves are shown in Figure 9A–D. Comparing Figure 9A,B and the simulation results of the tumor mouse model shown in Figure 6, the tumor-only model absorbs much lower doses under the same X-ray irradiation conditions than the tumor mouse model with body tissue beneath the tumor. Some tumors in mice demonstrate almost four to five times less absorption. The relationship between the DER and depth in models C and D with different kVp X-rays is shown in Figure 10. The DER is larger when surrounding tissue is present.

### 3.4. Comparison of the Simulations and Experiments

**Figure 11:**
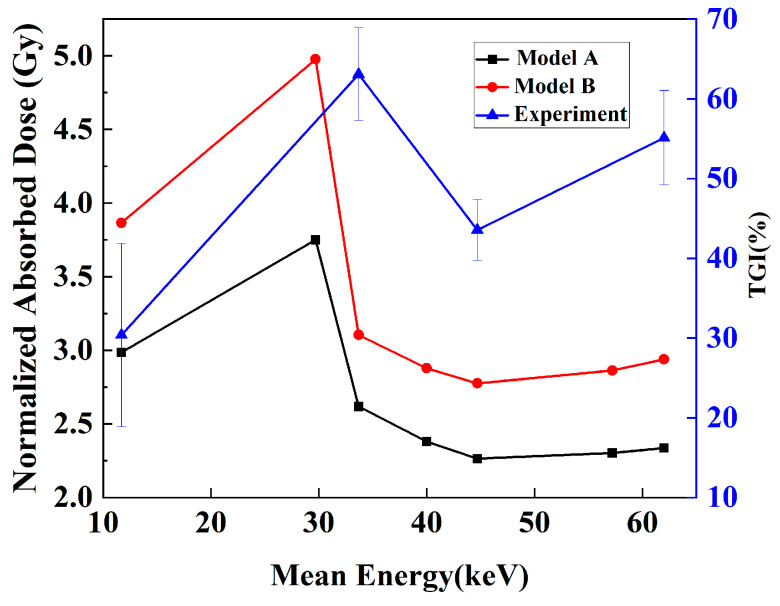
TGI in the experiment and the average absorbed dose (AAD) in simulation vs. mean energy of kVp X-rays. The TGI curve is represented by a blue line with triangles, whereas the AAD curve is represented by a black line with square points for Model A and a red line with dots for Model B.

The changes in the TGI with different kVps were experimentally obtained, as shown by the blue line in Figure 11. The results demonstrated that with the same incident dosage, the antitumor effect differed for different kVps. To compare the simulation results and the experiments at the same dosage level, the simulated doses were normalized according to the dose, and the AADs in the whole tumor of Models A and B were calculated for analysis. Figure 10 shows the AADs as a function of the mean energy under X-ray kVp. These results indicated that different X-ray mean energies had different AADs. This could lead to different TGIs. In both models, the AAD of X-rays initially increased and then decreased, and an evident peak at 29.7 keV (60 kVp) was formed; these results indicated that at this mean energy, the X-ray energy deposition was the highest. A comparison of the TGI and AAD curves revealed that the change trend of the AAD curve in Model B was more similar to that of the TGI curve. The similarity was evaluated with the Pearson correlation coefficient between the two indices. These coefficients were 0.68 and 0.75 for Models A and B, respectively.

To conduct a comprehensive quantitative assessment of the simulation results, the key performance indicators were calculated for each tube voltage and summarized in Table 1. The statistical quality of the simulation was very stable, remaining below 3.10% in all cases. The consistency between the simulated nanoparticle dose distribution (Model B) and the reference experimental data was excellent, with AAD always ranging from 1.2% to 1.3%. The dose enhancement effect quantified by DER was evident at all energy levels. It is noteworthy that at 60 kVp, DER reached its maximum value of 1.36, indicating that this is the most effective energy for achieving dose enhancement under the simulation conditions.

## 4. Discussion

Our simulation aims to conduct a quantitative analysis of the physical processes of X-PDT: the enhancement of local energy deposition. We fully acknowledge that physical dose enhancement does not singularly determine the final therapeutic outcome, as this involves critical biological factors, including photosensitizer efficiency or oxygen availability, not explicitly modeled here. However, our central hypothesis is that the physical dose enhancement serves as a key factor for the initial yield of ROS.

The dose and dose enhancement by the use of a certain X-PDT nanoparticle were examined to determine the optimum X-ray kVp (representing different X-ray energies) using MC simulations. This enhancement was determined by calculating the DER using physical tumor models with and without nanoparticles. When the models were irradiated by X-rays in the range of 20–160 kVp, the highest DER was obtained at 60 kVp; these results indicated that 60 kVp was the optimal X-ray kVp under the current simulation conditions because the lowest dose was needed to stimulate the X-PDT probe. The simulation method provides a potential way to optimize the irradiation conditions and treatment protocols for X-PDT.

The 3D dose distributions (Figure 5) were relatively spatially uniform. PDD curves (Figure 7) showed that for models without nanoparticles, above 60 kVp, absorbed dose decreased with depth; these results are similar to the PDD curves of 120 kVp and 220 kVp in the literature [20]. However, below 60 kVp, dose peaked at <1.0 mm depth before declining due to beam attenuation—confirming keV-level X-rays are suitable for superficial tumor treatment. For models with nanoparticles (Figure 6), peak doses were slightly higher for low kVp, while 80–160 kVp exhibited more pronounced dose peaks. This reflects a “dose-freezing effect” of the probe, which helps concentrate dose at the tumor site.

DER results (Figure 8) increased initially, stabilized, then decreased with depth for all kVp. Within 4.5 mm, DER exceeded 1.0 (maximum ~1.36), confirming the probe’s radiosensitizing effect—attributed to the photoelectric effect of high-Z elements in the probe [23,30]. While DER can be further improved by optimizing nanoparticle concentration or preparation processes [30], the sharp DER drop below 4.5 mm (<1.0) is due to the “shielding effect” of heavy metal nanoparticles (consistent with previous studies [19]), which protects normal organs and guides clinical application. The peak DER at 60 kVp (Figure 8) confirms its superior dose enhancement efficacy. To verify this, we estimated energy deposition in 5 mm-thick Lu using the mass energy absorption coefficient and X-ray spectrum (formula $D=\sum N_{i}\times E_{i}-\sum N_{i}\times E_{i}\times e^{-\mu t}$). The maximum absorbed dose at 60 kVp validated its optimal energy deposition.

When comparing the results of Model A and Model B to the tumor-only model in Figure 9 and Figure 10, both the doses and DER values were much greater when tissue was around the tumor. This was mainly caused by the combined effects of scattered radiation, the dose-building effect and backscattering in the surrounding tissues. Specifically, the presence of surrounding tissue significantly increased dose deposition in the tumor area, whereas when the tumor was simulated alone, the dose absorption was significantly reduced because of the lack of these effects. From a radiotherapy perspective, Models A and B simulated the scenario where tissues were present beneath the tumor, with X-rays irradiated from top to bottom; this process resembled the vertical irradiation approach commonly used in clinical practice. In contrast, Models C and D simulated the irradiation of an isolated tumor, which could be idealized as side irradiation of the tumor, thereby avoiding the influence of surrounding tissues. This comparison revealed the significant impact of the irradiation angle on the dose distribution: in vertical irradiation, the scattering and backscattering effects from surrounding tissues significantly enhanced the dose deposition in the tumor region, whereas in side irradiation, the reduced influence of surrounding tissues led to a more concentrated dose distribution but potentially lacked the dose enhancement effects contributed by the surrounding tissues. Therefore, in practical radiotherapy, optimizing the irradiation angle could be employed to adjust the dose distribution, thereby maximizing tumor dose deposition while minimizing damage to surrounding healthy tissues. Combining the radiosensitization and protective effect of the probes mentioned above, the introduction of the probe can effectively balance these two effects. Our results further illustrate the importance of probes for dose modulation in X-PDT.

A key finding of this study is the encouraging correlation observed between the simulated absorbed dose and the experimental TGI rates. While a formal statistical analysis with confidence intervals was not conducted due to the limited number of experimental data points, the qualitative trend alignment supports our central hypothesis. Specifically, our simulation predicted an optimal DER at 60 kVp, whereas the experimental TGI peaked at 80 kVp. This discrepancy is insightful and can be attributed to several factors absent in our idealized physical model. First, the experimental optimum was identified from a limited set of discrete energy settings; a denser sampling might yield a result closer to the simulated 60 kVp peak. Second, the simulation provides continuous, fine-grained data across the energy spectrum, effectively bridging the gaps between experimental measurements. Third, and most fundamentally, the DER primarily characterizes the physical radiosensitization component of the therapy. However, the overall efficacy of X-PDT is a multi-modal effect that is also critically dependent on tumor oxygenation status and singlet oxygen yield; these factors are inherent to the photodynamic process but do not exist in a purely physical dose model. Consequently, it is understandable that the correlation of the coefficient between the simulated dose and the experimental TGI is not strong. This study provides a valuable quantitative reference for dissecting the radiotherapy-related contribution within the complex anti-tumor mechanisms of X-PDT. Future studies should aim to incorporate these physiological and chemical variables to establish a more comprehensive predictive model.

While our findings establish an encouraging correlation between the simulated physical metrics and experimental outcomes, it is crucial to acknowledge the limitations of the current framework. Our simulation employed a simplified model consisting of a homogeneous water phantom with a uniform nanoparticle distribution. This idealization does not account for complex clinical realities such as tissue-induced beam hardening, which could shift the optimal incident kVp, or heterogeneous nanoparticle accumulation, which would lead to spatially varying dose enhancement and likely cause an overestimation of the average therapeutic effect. Additionally, this uniform model simplifies the discrete nature of NPs. Bulin et al. demonstrated that particle size affects the self-absorption effect of energy [14]. We also conducted corresponding supplementary verification, indicating that when the size of NPs is small, the self-absorption effect of NPs is relatively small. Therefore, the energy deposition is approximately that of a continuous medium, which provides a basis for our average dose method. Indeed, our own simulations under non-uniform conditions confirm this, demonstrating that the spatial distribution of the probes profoundly impacts the resulting dose deposition pattern. Furthermore, our study was confined to the diagnostic and low-energy therapeutic range, such as 20–160 kVp, which is characterized by limited tissue penetration depth. Therefore, our conclusions on optimal energy may not be directly translatable to deep-seated tumors or treatments involving higher-energy sources like MeV photons, where Compton scattering becomes the dominant interaction mechanism. Most fundamentally, our in silico framework lacks key biological determinants of tumor growth inhibition, including nanoparticle pharmacokinetics, cellular uptake, tumor hypoxia, and subsequent host immune responses. Consequently, the comparison with experimental data remains primarily qualitative, and the model cannot provide a quantitative prediction of the final therapeutic outcome. In light of these limitations, this model should be regarded as a powerful tool for optimizing the initial physical and chemical events of X-PDT, with the observed consistency serving as an encouraging verification of the underlying mechanism, rather than a quantitative prediction of the final therapeutic outcome. Future work focused on coupling our physical model with modules that account for patient-specific anatomies and these complex biological factors, along with validation in clinically relevant large animal models, is essential for advancing toward a truly predictive clinical tool.

Nevertheless, our findings offer several actionable insights that can directly guide the design of clinical protocols, even at this early stage. First, the identification of an optimal X-ray energy range provides a rational, physics-based starting point for selecting the irradiation source in clinical trials, moving beyond empirical approaches. Second, our simulation platform serves as a rapid and cost-effective pre-clinical screening tool to compare the relative efficacy of different nanoparticle candidates (e.g., varying material, size, or concentration), thereby prioritizing the most promising agents for experimental validation. Finally, the calculated dose enhancement factors, while idealized, can inform initial dosimetry considerations and help formulate hypotheses for treatment planning in early-phase studies. In essence, this computational framework acts as a translational bridge, helping to de-risk and optimize key physical parameters before they are tested in a clinical setting.

## 5. Conclusions

In summary, the dose-sensitization effect of kV X-ray energy on X-PDT was thoroughly examined using the MC simulation method. The optimized X-ray kVp of 60 was determined, and the results demonstrated its feasibility for optimizing the X-PDT treatment protocols. Although the proposed method achieved remarkable results in the study of the dose-sensitization effects of X-PDT, several limitations remain. First, insufficient conditions were simulated to fully cover all factors that could affect the effect of X-PDT. Second, X-ray-excited PDT efficiency was not considered in this study and could affect the comprehensiveness and accuracy of the results. To further refine and optimize the method, future studies will aim to simulate more complex conditions, such as a wider energy range and different tissue types and structures, to more fully evaluate the dose-sensitization effects of X-PDT. Moreover, the proportion of the PDT effect along with the dose enhancement will be further examined to improve the accuracy and practicability of the method.

## Figures and Tables

**Table 1:** Key Metrics for Simulated Dose Profiles, including Average Absolute Deviation (AAD), Peak Dose Enhancement Ratio (DER), and Maximum Relative Uncertainty at Various Tube Voltages.

Tube Voltage (kVp)	AAD	Dose Enhancement (Peak DER)	Maximum Relative Uncertainty
20	1.1789	1.22695	3.10%
40	1.29464	1.33981	2.83%
60	1.32702	1.3602	2.79%
80	1.18562	1.22197	2.96%
100	1.20931	1.25181	1.40%
120	1.2259	1.27113	2.43%
140	1.24356	1.28966	1.67%
160	1.25827	1.30478	1.55%

Note: The correlation coefficients were 0.68 and 0.75 for Models A and B, respectively.

## Data Availability

No new data were created or analyzed in this study. Data sharing is not applicable to this article.
